# A Turn-On Lipid Droplet-Targeted Near-Infrared Fluorescent Probe with a Large Stokes Shift for Detection of Intracellular Carboxylesterases and Cell Viability Imaging

**DOI:** 10.3390/molecules28052317

**Published:** 2023-03-02

**Authors:** Chenglin Li, Sifan Li, Xinsheng Li, Tao Yuan, Jialei Xu, Xixin Gu, Jianli Hua

**Affiliations:** Key Laboratory for Advanced Materials, Joint International Research Laboratory for Precision Chemistry and Molecular Engineering, School of Chemistry and Molecular Engineering, East China University of Science and Technology, 130 Meilong Road, Shanghai 200237, China

**Keywords:** fluorescent probe, large Stokes shift, near-infrared emission, carboxylesterases detection, lipid droplets targeting

## Abstract

Carboxylesterases (CEs) play important physiological roles in the human body and are involved in numerous cellular processes. Monitoring CEs activity has great potential for the rapid diagnosis of malignant tumors and multiple diseases. Herein, we developed a new phenazine-based “turn-on” fluorescent probe DBPpys by introducing 4-bromomethyl-phenyl acetate to DBPpy, which can selectively detect CEs with a low detection limit (9.38 × 10^−5^ U/mL) and a large Stokes shift (more than 250 nm) in vitro. In addition, DBPpys can also be converted into DBPpy by carboxylesterase in HeLa cells and localized in lipid droplets (LDs), emitting bright near-infrared fluorescence under the irradiation of white light. Moreover, we achieved the detection of cell health status by measuring the intensity of NIR fluorescence after co-incubation of DBPpys with H_2_O_2_-pretreated HeLa cells, indicating that DBPpys has great potential applications for assessing CEs activity and cellular health.

## 1. Introduction

As significant members of the serine hydrolase superfamily [1], carboxylesterases (CEs) catalyze the cleavage of esters of substrates into the corresponding alcohols and carboxylic acids in living organisms and are intimately involved in various metabolic and transport processes in the human body (e.g., protein metabolism, ester metabolism, detoxification, gene expression and biological signaling) [2,3,4]. Studies have shown that an abnormal expression of CEs is closely associated with many diseases such as malignancy, hyperlipidemia, atherosclerosis, Wolman disease and diabetes mellitus [5,6]. Other than being involved in biological metabolism, the targeting, delivery and release of some prodrugs are also associated with intracellular CEs activity [7]. More importantly, as one of the important biomarkers of cell metabolism, CEs play an important role in cell viability and cytotoxicity assays [8]. Therefore, the development of a reliable assay method to detect CE levels and thus reflect cellular status is much needed.

Lipid droplets (LDs), as one of the vital hubs in various intracellular physiological processes, are widely present in all types of mammalian cells [9,10]. Once thought to be merely inert fat particles, they are now considered to be highly dynamic, mobile organelles that interact and cooperate closely with a variety of cellular organelles [11]. Recent studies have shown that LDs are elevated in the hypoxic environment of tumors, giving LDs the potential for application in cancer diagnosis and treatment [12]. As one of the latest hot spots in the field of bioimaging, fluorescent probes targeting lipid droplets are also emerging. Researchers have developed a variety of fluorophore-based fluorescent probes for intracellular lipid droplet imaging [13,14,15]. However, there are still relatively few fluorescent probes that combine endogenous material detection and lipid droplet imaging.

Fluorescent probes are widely used because of their non-invasive nature, high sensitivity, low cost and real-time imaging [16,17]. Fluorescent probes are more suitable for the detection of biological endogenous molecules due to their better interference immunity, lower detection limits and more pronounced changes than colorimetric probes that vary in absorption. After reaction with the target molecule, fluorescent probes show changes in the fluorescence signal, such as wavelength, emission intensity and lifetime [18,19,20,21]. By tracking these changes, a number of fluorescent probes have been reported for the detection of CEs activity [13,22,23,24]. For example, Tang and Liu et al. [25] reported a fluorescent light-up probe for the specific detection of lysosomal esterase, but its emission wavelength was less than 540 nm. Our group previously reported a new diketopyrrolopyrrole-based ratiometric fluorescent probe for intracellular esterase detection, but its fluorescence emission could not reach the near-infrared region and the Stokes shift was small [26]. Compared to conventional fluorescent probes, near-infrared (NIR, 650–900 nm) fluorescent probes exhibit extraordinary advantages, such as deeper penetration in tissues, elimination of interference from background autofluorescence and great facilitation of the imaging of molecular processes in vivo [27,28,29,30,31]. Meanwhile, the large Stokes shift facilitates bioimaging by avoiding significant overlap between excitation and fluorescence spectra, as well as self-quenching caused by backscattering of biological samples [32,33].

Proverbially, the construction of donor–acceptor (D-A) structures has been considered an effective method for broadening the absorption/emission wavelengths of fluorescent probes [34,35,36]. Reduced phenazine as a typical donor (D) has the advantages of strong electron-donating ability, multiple modification sites, excellent stability and low biotoxicity [37,38,39]. In this paper, by introducing *p*-cyanopyridine to phenazine as an acceptor (A), we designed a new D-A fluorescent fluorophore, DBPpy, with NIR emission and large Stokes shifts of more than 250 nm. Benefiting from its strong hydrophobicity, DBPpy could locate in subcellular organelle lipid droplets (LDs).

**Scheme 1:**
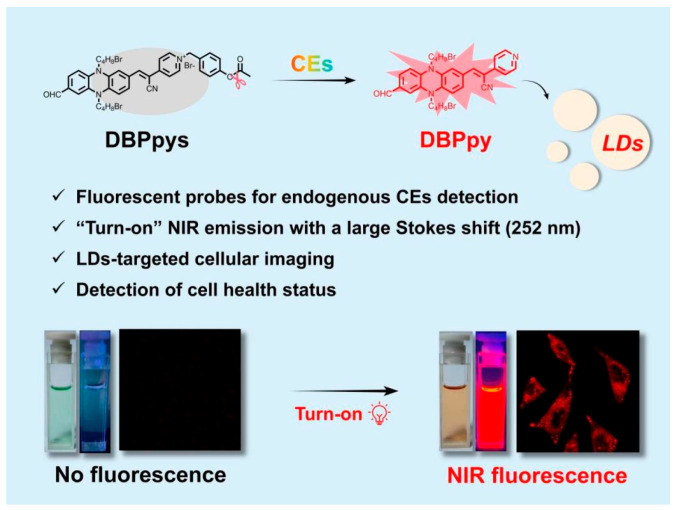
Schematic representation of the possible response mechanisms of fluorescent probe DBPpys activated by CEs.

Based on previous works [26,40,41], 4-bromomethyl phenyl ester has been commonly used for the recognition of CEs. Herein, we attached the moiety to the DBPpy to form a “turn-on” fluorescent probe DBPpys (Scheme 1). In the presence of various interfering substances, DBPpys still presented a specific reaction to CEs with a low LOD of 9.38 × 10^−5^ U/mL. Furthermore, the probe DBPpys was able to detect endogenous CEs activity in HeLa cells. After pretreating with H_2_O_2_, the viability of HeLa cells could be detected by changes in fluorescence, indicating that DBPpys has great potential applications for assessing CEs activity and cellular health.

## 2. Results and Discussion

### 2.1. Synthesis and Photophysical Properties

The synthetic route for DBPpys is shown in Scheme S1. First, the compound DBP-CHO was synthesized according to our previous literatures [42,43]. In the next step, *p*-cyanopyridine was introduced to DBP-CHO via the Knoevenagel reaction. Finally, the NIR fluorescent probe DBPpys was obtained via the electrophilic substitution of DBPpy and 4-bromomethyl phenyl ester. The detailed synthesis steps and reaction conditions were presented in the Supporting Information, and the important intermediates and target compounds were well characterized by ^1^H nuclear magnetic resonance (NMR) and ^13^C NMR spectroscopies and high-resolution mass spectrometry (HRMS) (Figures S8–S17).

**Figure 1:**
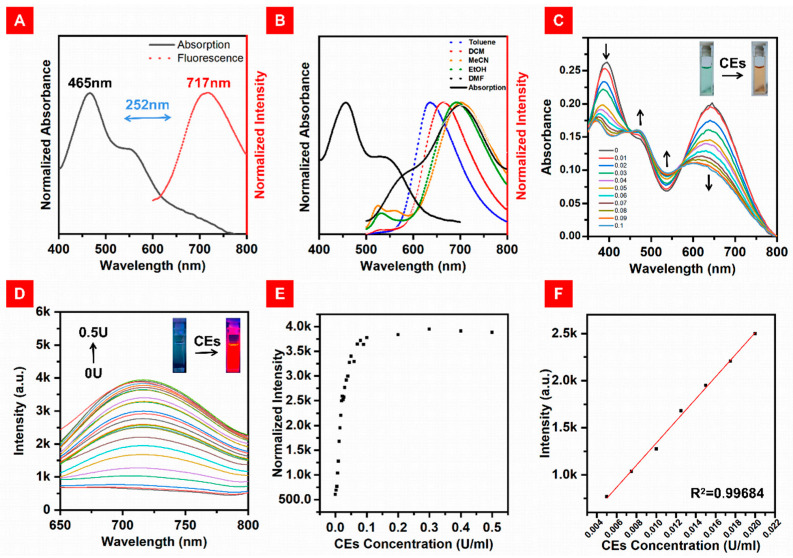
(**A**) Normalized absorption spectra (black solid line) and fluorescence spectra (red dotted line) of DBPpy in DMF:HEPES (*v:v* = 4:6, pH = 7.4). (**B**) Normalized fluorescence spectra (dotted lines) of DBPpy in different solvents and the absorption spectrum (solid line) of DBPpy in DMF. (**C**) Absorption spectra of DBPpys (10 μM) toward different concentrations of CEs in DMF:HEPES (*v:v* = 4:6, pH = 7.4). (**D**) Fluorescence spectra of DBPpys (10 μM) toward the concentrations of CEs from 0 U/mL to 0.5 U/mL in DMF:HEPES (*v:v* = 4:6, pH = 7.4). (**E**) Fluorescence intensity of DBPpys (10 μM) at 720 nm after reaction with the concentrations of CEs from 0 U/mL to 0.5 U/mL in DMF:HEPES (*v:v* = 4:6, pH = 7.4). (**F**) Linear fitting curve of the fluorescence intensity of DBPpys at 720 nm (10 μM) toward the concentrations of CEs from 0.005 U/mL to 0.02 U/mL.

After obtaining the target compounds, we tested their photophysical properties. As shown in Figure 1A, the maximum absorption peak of DBPpy was at 465 nm, together with a shoulder peak at 550 nm in mixed solvent (DMF:HEPES, *v:v* = 4:6). Additionally, an NIR emission at 717 nm was observed, and the fluorescence quantum yield of DBPpy was 4.8%. The Stokes shift of DBPpy was more than 250 nm in this mixed solvent, which is favorable for confocal imaging. We speculate that the large Stokes shift is facilitated by the donor–acceptor (D-A) structure’s intermolecular charge transfer (ICT) process from the phenazine donor (D) to the cyanopyridine electron acceptor (A). It is known that the ICT process is more likely to be influenced by the polarity of the solvent [44,45]. As shown in Figure S1B, the absorption spectra of DBPpy were similar in different solvents, which indicated that no conformational transition occurred at the ground state. However, the fluorescence emission spectra of DBPpy varied considerably with the different solvents. In nonpolar solvents such as toluene, the emission peak of DBPpy was located at 635 nm, while it was redshifted to 710 nm in DMF. With increasing polarity, the fluorescence redshifted and the emission intensity gradually decreased (as shown in Figure 1B and Figure S1A), which implied that ICT may have occurred.

### 2.2. Spectroscopic Response of DBPpys to CEs

Primarily, the response time of DBPpys towards CEs was measured in DMF:HEPES (*v:v* = 4: 6, pH = 7.4) at 37.4 °C. The fluorescence response of 10 μM DBPpys to 0.08 U/mL CEs during 0–90 min is shown in Figure S2. The fluorescence intensity increased significantly at 720 nm with increasing reaction time and leveled off after 60 min, indicating that the reaction could be completed within 60 min. Subsequently, the response of 10 μM DBPpys to different concentrations of CEs was tested after 60 min. After the reaction of DBPpys with CEs in this solvent mixture, the probe showed a clear color change from green to brown, and the intensity of the absorption peak at 400 and 690 nm decreased while that of the peak at 465 and 550 nm increased (as shown by the arrow in Figure 1C). Meanwhile, the fluorescence intensity at 720 nm gradually increased with the addition of CE equivalents in DBPpys solution (Figure 1D,F), which was consistent with the emission peak of DBPpy, indicating that the pyridine cation of DBPpys might turn into pyridine after the reaction with CEs. Interestingly, the fluorescence intensity at 720 nm in the range from 0.005 U/mL to 0.02 U/mL also showed a good linear relationship with the concentration of CEs with an equation of R_1_ = 117,260 × *C* (U/mL) + 164.66245 (R^2^ = 0.99684) (Figure 1G), suggesting that DBPpys can be used for endogenous imaging of CEs. The detection limit was calculated as 9.38 × 10^−5^ U/mL based on 3σ/*k*, where σ is the standard deviation of the blank measurement and *k* is the slope of the linear equation.

### 2.3. Exploration of Reaction Mechanism

**Figure 2:**
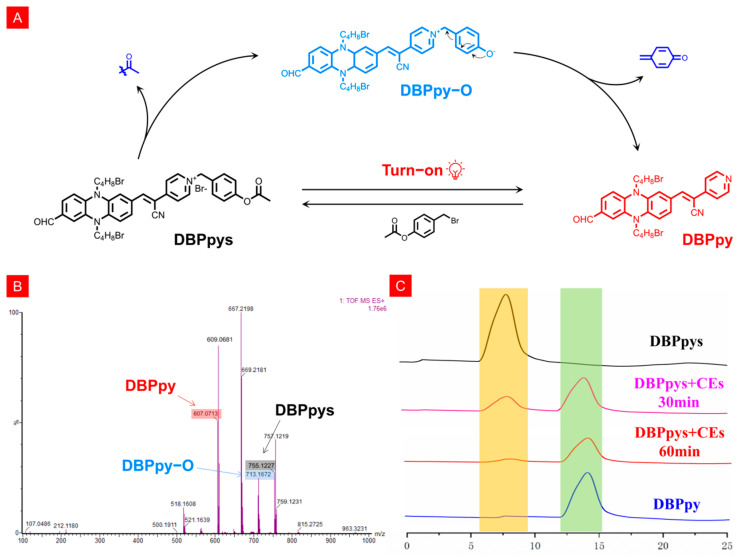
(**A**) Possible mechanism of DBPpys response to CEs. (**B**) HRMS of DBPpys before and after reaction with CEs. (**C**) HPLC analysis of DBPpys after reaction with CEs at different times.

Since the fluorescence emission spectrum of the probe DBPpys after the addition of CEs was essentially the same as that of DBPpy, we hypothesized that the reaction mechanism was the specific excision of the acetate group by CEs, which led to the departure of the benzene ring adjacent to phenazine in the quinone form, thus returning to the structure of DBPpy, and the NIR fluorescence was eventually restored (Figure 2A). To verify this idea, we performed mass spectroscopy and high-performance liquid chromatography (HPLC) analysis. After extraction with dichloromethane, the high-resolution mass spectra of DBPpys with CEs were measured (Figure 2B). The mass fragments observed at 834.0416 and 606.0630 belonged to DBPpys ([M]_cal_^+^ = 755.1227) and DBPpy ([M + H]_cal_^+^ = 607.0708). Fortunately, we found the ion peak of the putative reaction intermediate DBPpy−O ([M + H]_cal_^+^ = 713.1121) in the mass spectrum, clearly validating the proposed mechanism. As shown in Figure 2C, the retention times of DBPpys and DBPpy were 8.1 and 14.4 min, respectively. When CEs was added to DBPpys solution, the 8.1 min peak belonging to DBPpys decreased, and a new peak at 14.4 min close to that of DBPpy appeared.

### 2.4. Temperature and pH Effect on the Probe and Selectivity

**Figure 3:**
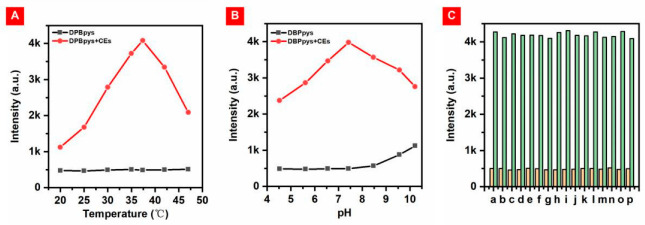
(**A**) Fluorescence intensity at 720 nm of DBPpys (10 μM) with (red point) and without (black point) CEs in DMF:HEPES (*v*:*v* = 4:6) at different temperatures. (**B**) Fluorescence intensity of DBPpys (10 μM) at 720 nm with (red point) and without (black point) CEs in DMF:HEPES (*v*:*v* = 4:6) with different pH values. (**C**) Fluorescence intensity of DBPpys (10 μM) at 720 nm before (orange columns) and after (green columns) reaction with CEs in the presence of 200 μM of various interfering substances in DMF*:*HEPES (*v*:*v* = 4:6, pH = 7.4) at 37.4 °C. a: blank; b: Na_2_CO_3_; c: Na_2_SO_4_; d: KCl; e: H_2_O_2_; f: ClO^−^; g: Hcy; h: Cys; i: GSH; j: ADP; k: ATP; l: BSA; m: LAP; n: HSA; o: AFP; p: APN.

In order to test whether DBPpys can be used for the detection of CEs in a biological environment, the performance of DBPpys in reaction with CEs under different temperatures and pH conditions was investigated. The probe DBPpys showed almost no fluorescence in the absence of CEs, and the fluorescence intensity of DBPpys increased with the addition of CEs under normal human body temperature and pH conditions (as shown in Figure 3A,B). More importantly, the NIR fluorescence emission was significantly enhanced with the addition of CEs at 37.4 °C and pH = 7.4, close to the biological environment. This result indicates that the probe can be effectively detected in a physiological environment.

The chemical composition in the physiological environment is more complex, and probe DBPpys requires good selectivity for its application in living organisms. We measured the changes in emission intensity before and after the reaction of DBPpys with CEs after the addition of different interfering substances, including inorganic salts (Na_2_CO_3_, Na_2_SO_4_ and KCl), reactive oxygen species (ROS) (H_2_O_2_ and ClO^−^), three common amino acids (Hcy, Cys and reduced glutathione (GSH)), adenosine 5′-diphosphate (ADP), adenosine 5′-triphosphate (ATP) and proteins (bovine serum albumin (BSA), leucine aminopeptidase (LAP), human serum albumin (HSA), α-fetoprotein (AFP) and aminopeptidase N (APN)). DBPpys exhibited excellent specificity for CEs despite the presence of different interfering substances (Figure 3C). This indicates that DBPpys can be well applied in biological environments and is expected to be used for endogenous imaging of CEs in cells.

### 2.5. Intracellular Endogenous CE Detection

As discussed above, DBPpys could assess CEs activity in vitro. The applications of DBPpys in living cells were further examined. First, the cytotoxicity of DBPpys was evaluated by CCK-8. DBPpys showed low cytotoxicity to HeLa cells, with cell survival exceeding 90% after 24 h of incubation at a concentration of 40 μM, indicating that DBPpys had little effect on cells at the working concentration (10 μM) and is suitable for application in biological systems (Figure S3).

**Figure 4:**
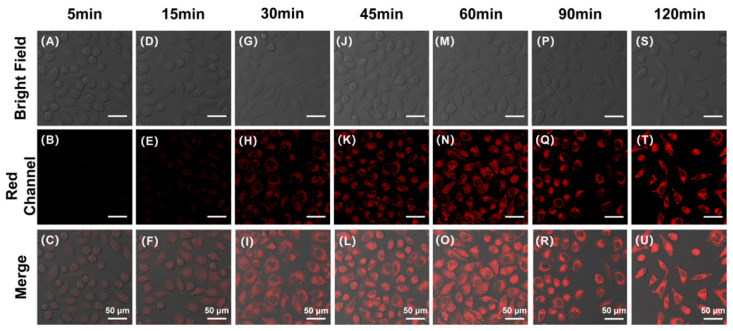
Fluorescent imaging of HeLa cells stained with 10 μM DBPpys at different incubation times. Incubation times: (**A**–**C**) 5 min; (**D**–**F**) 15 min; (**G**–**I**) 30 min; (**J**–**L**) 45 min; (**M**–**O**) 60 min; (**P**–**R**) 90 min; (**S**–**U**) 120 min. (**A**,**D**,**G**,**J**,**M**,**P**,**S**) bright field; (**B**,**E**,**H**,**K**,**N**,**Q**,**T**) red channel (650–750 nm); (**C**,**F**,**I**,**L**,**O**,**R**,**U**) merge image. *λ*_ex_ = 550 nm. Scale bar = 50 μm.

To investigate the optimal co-incubation time of DBPpys with HeLa cells, as shown in Figure 4, we obtained fluorescence imaging maps after different reaction times by confocal laser scanning microscopy (CLSM). As can be seen from Figure 4A–C, HeLa cells co-incubated with DBPpys for 10 min already started to show fluorescence in the red channel. The fluorescence intensity increased with longer incubation times and reached its maximum when the incubation time was 60 min (Figure 4 and Figure S5A). Therefore, DBPpys has a good ability to respond to endogenous CEs in live cells; consequently, we chose 60 min as the best incubation time for DBPpys with HeLa cells.

Subsequently, to verify the effect of CEs activity on the imaging effect of DBPpys, a CEs inhibitor, 4-(2-aminoethyl)benzenesulfonyl fluoride hydrochloride (AEBSF), was selected for further study [46,47]. The toxicity of AEBSF was evaluated using CCK-8, and it was confirmed that AEBSF had little effect on cells at working concentrations (Figure S3C). As shown in Figure S5, NIR fluorescence intensity of the cellular red channel was high after co-incubation with DBPpys for 60 min when no or a small amount of AEBSF was added. In contrast, as shown in Figure S4B, as the AEBSF content continued to increase, NIR fluorescence intensity decreased sharply, and at 2 mM, the fluorescence was almost invisible to the naked eye. The signal changes confirmed the specific response of DBPpys to endogenous CEs in HeLa cells and indicated that it can distinguish the CEs activity in cells based on the magnitude of fluorescence intensity.

### 2.6. Detection of Healthy Status of Cells Pretreated with H_2_O_2_

Hydrogen peroxide (H_2_O_2_) is an intermediate product of cellular oxygen metabolism commonly found in aerobic organisms. Many studies have shown that H_2_O_2_, as a common biomarker of stable ROS and oxidative stress in organisms, has significant damaging effects on cells and tissues, being capable of affecting cell viability and even causing cellular damage and death. CEs viability will be significantly stronger in healthy HeLa cells than in damaged ones. It also offers the possibility of detecting the healthy status of cells by analyzing the activity of CEs. Consequently, we pretreated HeLa cells with different concentrations of H_2_O_2_ before incubation with DBPpys (10 μM) to test whether the probe DBPpys can accurately reveal the health status of the cells.

**Figure 5:**
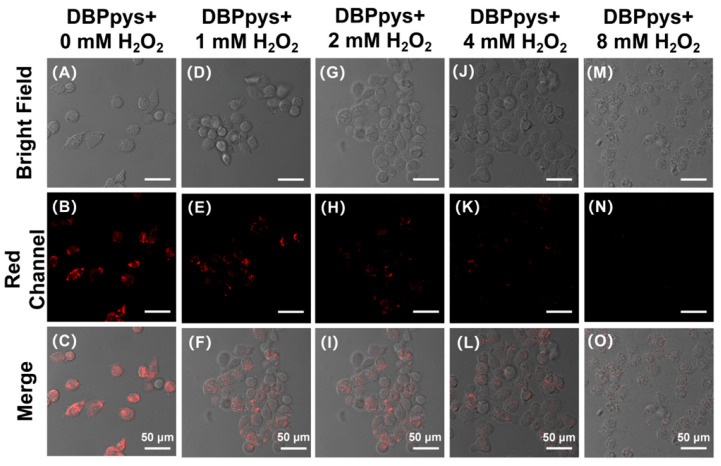
Fluorescent images of HeLa cells incubated with 10 μM DBPpys for 60 min after pretreating with different amounts of H_2_O_2_ for 2 h. H_2_O_2_ concentrations: (**A**–**C**) 0 mM; (**D**–**F**) 1 mM; (**G**–**I**) 2 mM; (**J**–**L**) 4 mM; (**M**–**O)** 8 mM. (**A**,**D**,**G**,**J**,**M**) bright field; (**B**,**E**,**H**,**K**,**N**) red channel (650–750 nm); (**C**,**F**,**I**,**L**,**O**) merge image. *λ*_ex_ = 550 nm. Scale bar = 50 μm.

As shown in Figure 5A–C, HeLa cells not pretreated with H_2_O_2_ showed a clear NIR emission signal in the red channel, indicating that CEs were highly active in the cells, thus proving that the state of the cells was hardly impaired. As the concentration of H_2_O_2_ pretreatment increased, NIR fluorescence gradually became weaker (as shown in Figure S5C), proving that CEs activity was restricted and the activity of the reaction with DBPpys was reduced, which was attributed to the gradual decrease in cellular activity after H_2_O_2_ stimulation. When the H_2_O_2_ pretreatment concentration reached 8 mM, NIR fluorescence almost disappeared and was no longer observable through the naked eye, and the bright-field images showed unhealthy cell status. These results strongly proved that DBPpys can be used to assess the health status of cells.

### 2.7. Intracellular Lipid Droplet Colocalization

Lipid droplets (LDs), a subcellular organelle capable of regulating cellular energy homeostasis, have received a lot of attention from researchers in recent years [48,49,50]. Since the fluorescence of DBPpys was severely quenched, we only needed to explore whether DBPpy could be used as a lipid droplet-targeted probe. The oil–water partitioning experiment (Figure S6) showed that DBPpy could be easily transferred from the lower aqueous phase to the upper oleic acid layer by simple oscillation, demonstrating its strong hydrophobicity. The ClogP value of DBPpy was 6.859, as calculated using Chemdraw Professional 16.0, whereas previous studies have shown that compounds usually have excellent LD-targeting ability when their ClogP values are higher than 5.0.

**Figure 6:**
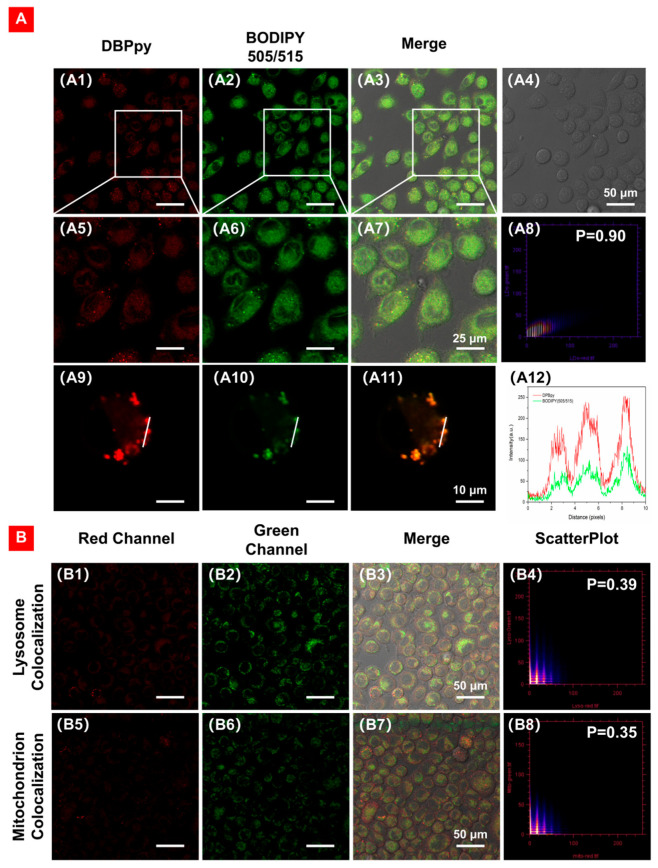
(**A**) CLSM of HeLa cells co-stained with (**A1**,**A5**,**A9**) DBPpy (10 μM, red channel), (**A2**,**A6**,**A10**) BODIPY 505/515 (5 µg·mL^−1^, green channel), (**A3**,**A7**,**A11**) overlay images of red channel, green channel and bright field, (**A4**) bright field, (**A8**) intensity correlation plot of BODIPY 505/515 and DBPpy, R = 0.90, and (**A12**) intensity profiles of ROI across a HeLa cell. *λ*_ex_: 550 nm; *λ*_em_: 650–750 nm for DBPpy (red); *λ*_ex_: 490 nm; *λ*_em_: 500–530 nm for commercial fluorophores (green). (**B**) Co–localization of DBPpy with lysosomes (**B1**–**B4**) and mitochondria (**B5**–**B8**).

Next, we further validated the LD-targeting ability of DBPpy by co-staining HeLa cells using the commercial LD-imaging agent BODIPY 505/515. As depicted in Figure 6A, the NIR fluorescence signal in the red channel is from DBPpy, and the fluorescence signal in the green channel is from the commercial dye BODIPY (505/515). It is evident that the two signals can overlap well, with a high Pearson correlation factor (R) of 0.90 and excellent consistency in changes in intensity profiles of regions of interest (ROI) (Figure 6(A8,A12)). To demonstrate the specific targeting ability of DBPpy on LDs, we also performed confocal experiments on other organelles, such as mitochondria and lysosomes. As shown in Figure 6B, the commercial lysosomal probe Lyso tracker Green DND-26 and the commercial mitochondrial probe Mito tracker Green FM were used to co-incubate with DBPpy in HeLa cells, but the Pearson coefficients were only 0.39 and 0.35, respectively. The results showed that DBPpy could target well in LDs, revealing that DBPpy was an excellent candidate for LD localization.

## 3. Experimental Section

### 3.1. Materials

All reagents were bought from commercial sources (Leyan (Beijing, China), Energy Chemical (Anqing, China), Sigma-Aldrich (Shanghai, China), Adamas-beta (Shanghai, China)) and used without further processing. All solvents were purified and dried before use by standard methods. The solvents used in spectrum analysis were HPLC-grade. The solutions for analytical studies were prepared with deionized water treated using a Milli-Q System (Billerica, MA, USA).

Human cervical carcinoma cells (HeLa cells) were purchased from Shanghai Xinyu Biotechnology Co., Ltd. (Shanghai, China). Green fluorescence dye BODIPY493/503 lipid droplets were purchased from GlpBio Co. (Montclair, CA, USA). LysoTracker Green DND 26 and MitoTracker Green FM were purchased from Life Technologies Co (Carlsbad, CA, USA). DMEM High Glucose w/L-Glutamine w/Sodium Pyruvate (DMEM), Fetal Bovine Serum (FBS) South America (FBS), Penicillin-Streptomycin Solution 100× and Trypsin 0.25%–EDTA 0.02% in HBSS were from Yuli Biotechnology Co., Ltd (Shanghai, China). Cell-Counting-Kit-8 (CCK-8) and 4-(2-Aminoethyl)-benzenesulfonyl fluoride hydrochloride (AEBSF) were from Aladdin (Shanghai, China).

### 3.2. Instruments

^1^H NMR and ^13^C NMR spectra were obtained with Bruker AM 400 MHz spectrometer and Ascend 600 MHz spectrometer using CDCl_3_ or DMSO-*d*_6_ as solvent, and tetramethylsilane (TMS) was used as an internal standard. Electrospray ionization and electron spray ionization were determined using Waters Micromass LCT mass spectrometer and Xevo G2 TOF MS. Absorption spectra were recorded on a Varian Cary 500 UV-vis spectrophotometer. Fluorescent spectra were recorded on a RF-6000 Fluorescence Spectrophotometer (SHIMADZU). Cell fluorescence images were captured using Leica Microsystems’ TCS SP5 II confocal fluorescence microscope.

### 3.3. Titration and Calculation of LOD

In a 1.0 mL cuvette containing DMF-HEPES solution (4:6, *v*/*v*), various equivalents of CEs were added to DBPpys (10 μM) at pH = 7.4. The mixtures were shaken at 37.4 °C for 30 min, and then fluorescence spectra were acquired with an excitation wavelength of 720 nm. A linear relationship in the range from 0.005 to 0.02 U was illustrated by the square of correlation coefficient equaling 0.99684. Based on the linear fitting in Figure 1F, the LOD was estimated with the following formula:

Limit of detection (LOD) = 3σ/k

where σ is the standard deviation of blank measurements (n = 11), and k is the slope of the linear fitting curve between the emission intensity at 720 nm and the concentration of CEs from 0.005 to 0.02 U, respectively.

### 3.4. Anti-Interference

Na_2_CO_3_, Na_2_SO_4_, KCl, H_2_O_2_, ClO^−^, Hcy, Cys, GSH, ADP, ATP, BSA, LAP, HSA, AFP and APN were obtained from commercial sources (Le Yan) and used without additional purification.

DBPpys (10 μM) reacted with interfering substances (200 μM) or CEs (0.1 U) for 30 min in a shaker at 37.4 °C.

### 3.5. Cell Cultures

The HeLa cells were propagated in T-25 flasks and cultured at 37 °C under a humidified 5% CO_2_ atmosphere in Dulbecco’s Modified Eagle Medium (DMEM) containing 10% heat-inactivated fetal bovine serum (Invitrogen, Calsbad, CA, USA) and 1% penicillin-streptomycin (10,000 U/mL penicillin and 10 mg/mL streptomycin).

### 3.6. Cytotoxicity Assay by CCK-8

The HeLa cells were seeded in 96-well plates and cultured in standard 0.2 mL DMEM medium containing 10% FBS (Invitrogen, Calsbad, CA, USA) and 1% antibiotics (penicillin, 10,000 U mL^−1^, and streptomycin, 10 mg mL^−1^) for 24 h (37 °C, 5% CO_2_). The DBPpys stock solution was diluted to required concentrations (0 μM, 2.5 μM, 5 μM, 10 μM, 20 μM and 40 μM) with culture medium and then the previous medium was replaced by the diluted solutions. After incubation for 24 h, absorbance was measured at 450 nm using a multifunctional microplate reader (Synergy H1, BioTek Instruments, Vermont, USA). The relative cell viability (%) was calculated by the following formula:

cell viability = ODtreated/ODcontrol × 100%

### 3.7. Intracellular CE Detection

The HeLa cells were seeded in confocal dishes and incubated for 24 h. The cells were co-incubated with DBPpys at a final concentration of 10 μM (containing 1% DMSO) and incubated for 5–360 min at 37 °C in an atmosphere of 5% CO_2_ and 95% air.

In the inhibition experiments, HeLa cells were first incubated with different concentrations of AEBSF (0.5 mM, 1 mM, 2 mM) for 30 min and then incubated with DBPpys (10 μM) for 60 min.

Fluorescence imaging was then performed using a confocal laser scanning microscope (CLSM, Leica Microsystems, TCS SP5 II, Wetzlar, Germany). The fluorescence signals of cells incubated with probes were collected at 650–750 nm, using a laser at 550 nm as excitation resource.

### 3.8. Cell Co-Localization Imaging

The HeLa cells were seeded in confocal dishes and incubated for 24 h. The cells were incubated with Mito Tracker Green FM (200 nM) or Lyso Tracker Green DND-26 (100 nM) for 30 min at 37 °C. The culture medium was removed, and cells were then washed twice with PBS and co-incubated with DBPpy (10 μM) for an additional 60 min.

## 4. Conclusions

In this work, we designed a NIR fluorophore DBPpy with a large Stokes shift (over 250 nm) by constructing a D-A structure, which could specifically localize in LDs. By introducing 4-bromomethyl phenyl ester to DBPpy, the fluorescent probe DBPpys for CEs was developed, which showed a “turn on” NIR fluorescence signal and a distinct color change from green to brown with a low detection limit of 9.38 × 10^−5^ U/mL in vitro. Additionally, in the presence of other analytes, DBPpys showed specific recognition of CEs. Moreover, endogenous CEs activity is closely related to cell viability. Therefore, the probe DBPpys was successfully used to analyze the survival status of cells pretreated with H_2_O_2_ by detecting the differences in fluorescent intensity. These results provide a strategy for the development of additional NIR fluorescent probes for CEs activity monitoring and a potential tool for in vivo fluorescence imaging.

## Data Availability

The data from this study are available from the corresponding author upon reasonable request.

## Supplementary Materials

The following supporting information can be downloaded at https://www.mdpi.com/article/10.3390/molecules28052317/s1, Figure S1: Fluorescence and absorption spectra of DBPpy in different solvents; Figure S2: Fluorescence spectra of DBPpys after different times of reaction with CEs; Figure S3: Cytotoxicity testing; Figure S4: CLSM of DBPpys after coincubation with HeLa cells after AEBSF pretreatment; Figure S5: Average fluorescence intensity of cells on CLSM; Figure S6: Oil–water distribution experiment; Section S1: Synthesis and characterization; Scheme S1: Synthetic route to DBPpys target compound; Figure S7–15: NMR and HRMS spectra. References [29,43] are cited in the supplementary materials.

## Sample Availability

Samples of some compounds may be available from the authors.
