# Supplementary material for: A Turn-On Lipid Droplet-Targeted Near-Infrared Fluorescent Probe with a Large Stokes Shift for Detection of Intracellular Carboxylesterases and Cell Viability Imaging
Source: Molecules. 2023 Mar 2;28(5):2317. doi: 10.3390/molecules28052317 (PMC10005208; doi:10.3390/molecules28052317)
Supplement: Supplementary file 1 [file molecules-28-02317-s001.zip › molecules-2097657-supplementary.pdf]

Electronic Supplementary Information (ESI)

A Turn-on Lipid Droplet-targeted Near-infrared Fluorescent

Probe with a Large Stokes Shift for Detection of Intracellular

Carboxylesterases and Cell Viability Imaging

Chenglin Li †, Sifan Li †, Xinsheng Li, Tao Yuan, Jialei Xu, Xixin Gu and Jianli Hua *

Key Laboratory for Advanced Materials, Joint International Research Laboratory for Precision Chemistry and Molecular Engineering, School of Chemistry and Molecular Engineering, East China University of Science and Technology, 130 Meilong Road, Shanghai 200237, China

* Correspondence: jlhua@ecust.edu.cn

† These authors contributed equally to this work.

Table of Contents

Section	Title	Pages
Figure S1	Fluorescence and absorption spectra of **DBPpy** in different solvents	S3
Figure S2	Fluorescence spectra of **DBPpys** after different times of reaction with CEs	S3
Figure S3	Stability testing	S4
Figure S4	pKa testing	S4
Figure S5	Cytotoxicity testing	S5
Figure S6	CLSM of DPBpys after coincubation with HeLa cells after AEBSF pretreatment	S5
Figure S7	Average fluorescence intensity of cells on CLSM	S6
Figure S8	Oil-water distribution experiment of **DBPpy**	S6
Section S1	Synthesis and characterization	S7
Scheme S1	Synthetic route to the target compound **DBPpys**	S7-S9
Figure S9-17	NMR and HRMS spectra	S9-S13
References		S14

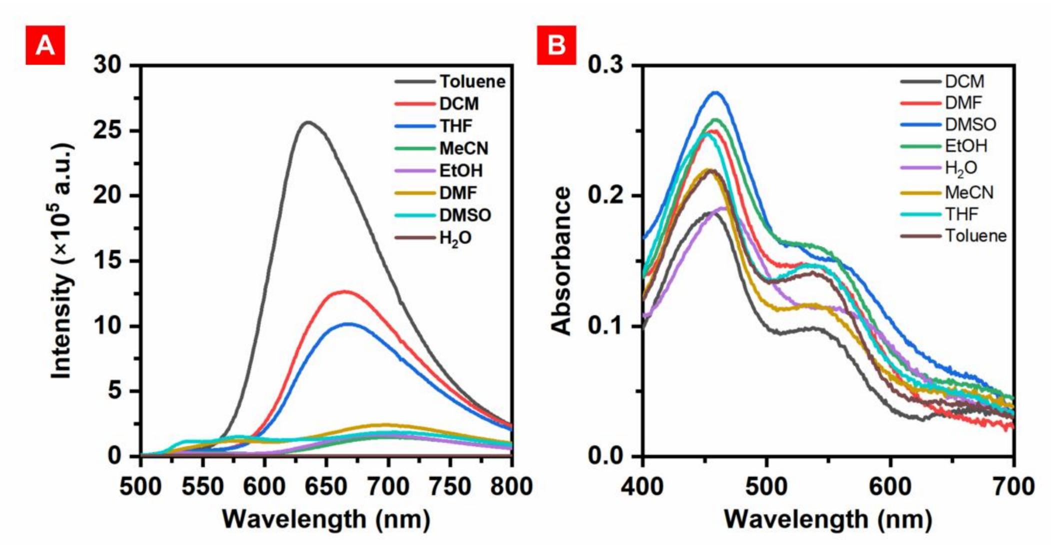

Figure S1. (A) Fluorescence spectra of **DBPpy** in different solvents. (B) Absorption spectra of **DBPpy** in different solvents.

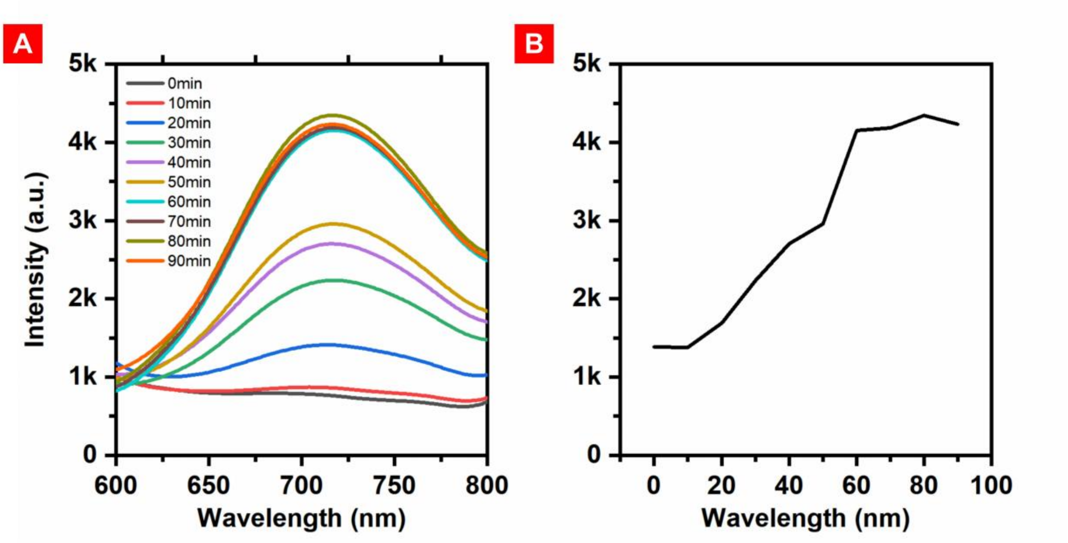

Figure S2. (A) Fluorescence spectra of **DBPpys** after different reaction times with CEs (10 μM) in DMF∶HEPES (v∶v = 4∶6, pH = 7.4). (**B**) The fluorescence intensity of **DBPpys** (10 μM) at 720 nm after different reaction times with CEs in DMF∶HEPES (v∶v = 4∶6, pH = 7.4).

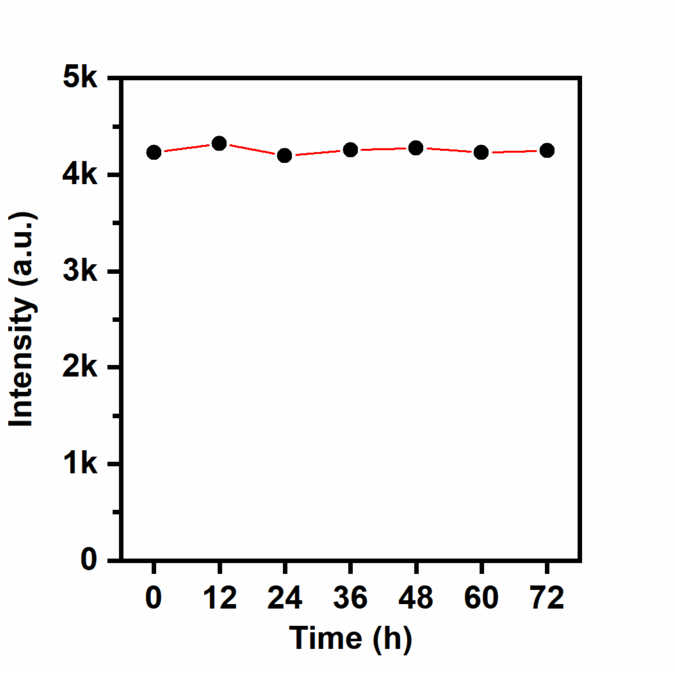

Figure S3. The plot of peak intensity of **DBPpy** (10 μM) at 720 nm in DMF : Cell Lysate (v : v = 4 : 6) at different time points.

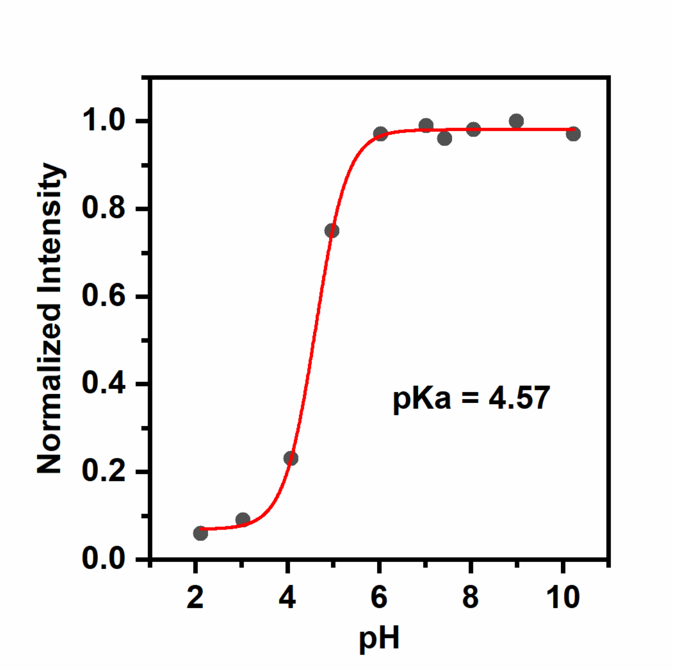

Figure S4. pH-dependent fluorescence intensity of **DBPpy** (10 μM in DMF : HEPES (v : v = 4 : 6) for various pH, monitored at 720 nm).

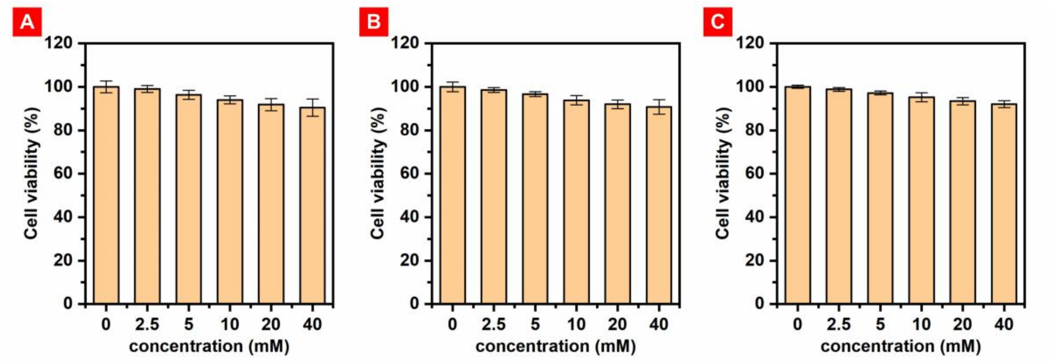

Figure S5. The viability of HeLa Cells after 24h of **DBPpys** (A), **DBPpy** (B) and AEBSF (C) treatment in the dark.

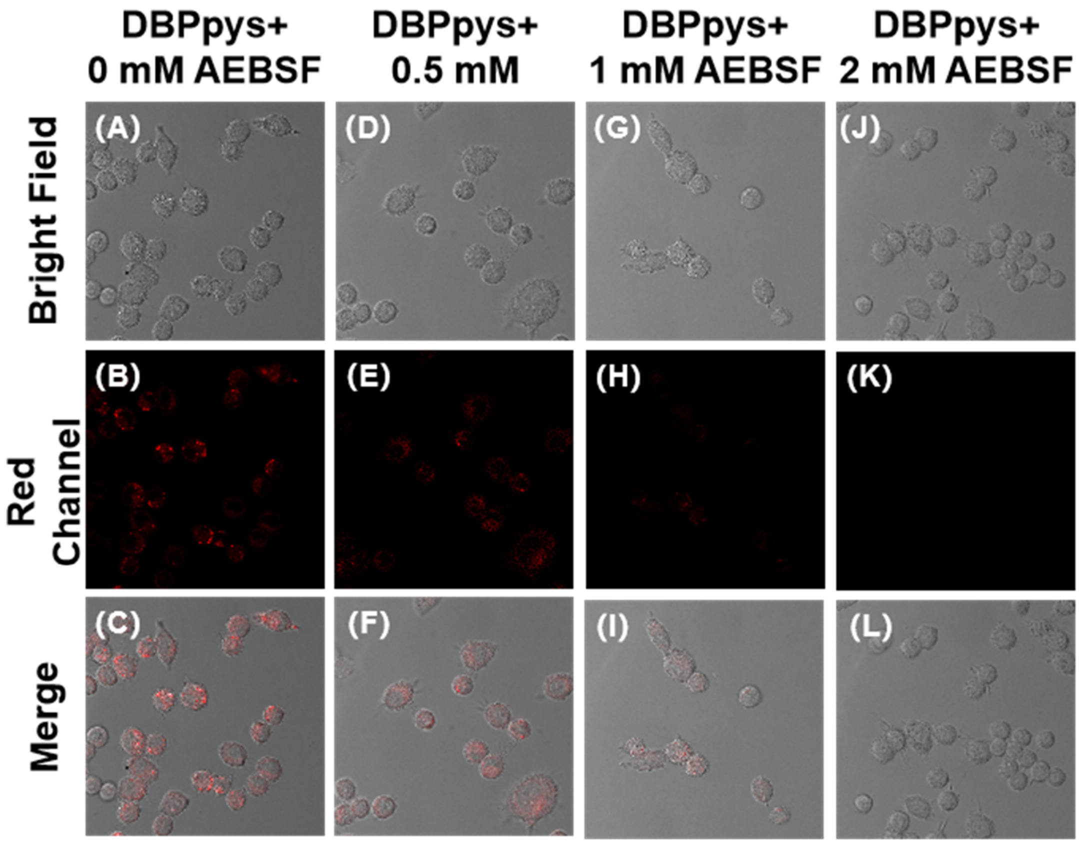

Figure S6. Fluorescent imaging of HeLa cells stained with 10 μM DBPpys after different AEBSF concentrations pretreatment. AEBSF concentration: (A-C) 0 mM; (D-F) 0.5 mM; (G-I) 1 mM; (J-L) 2 mM. (A, D, G, J) bright field; (B, E, H, K) red channel (650-750 nm); (C, F, I, L) merge image. λ_{ex} = 550 nm. Scale bar = 10 μm.

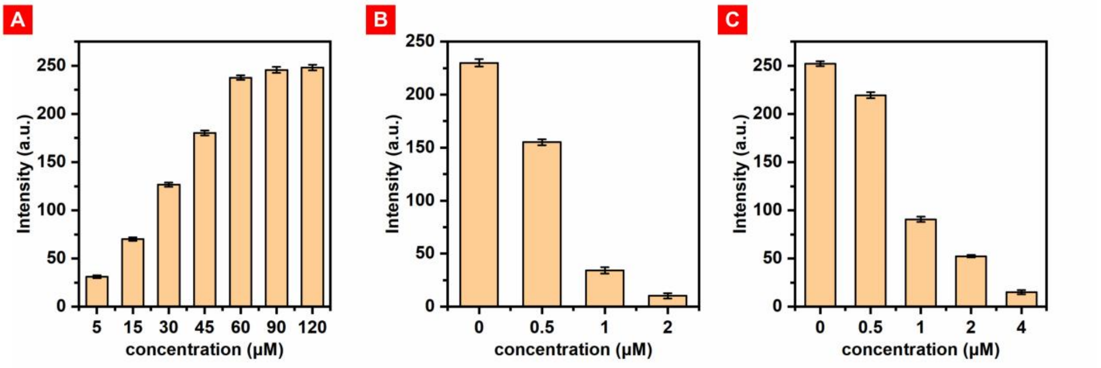

Figure S7. Average fluorescence intensity of HeLa cells (A) stained with 10 μM **DBPpys** with different incubation times; (B) stained with 10 μM **DBPpys** after different AEBSF concentrations pretreatment; (C) stained with 10 μM **DBPpys** after different H_2O_2 concentrations pretreatment.

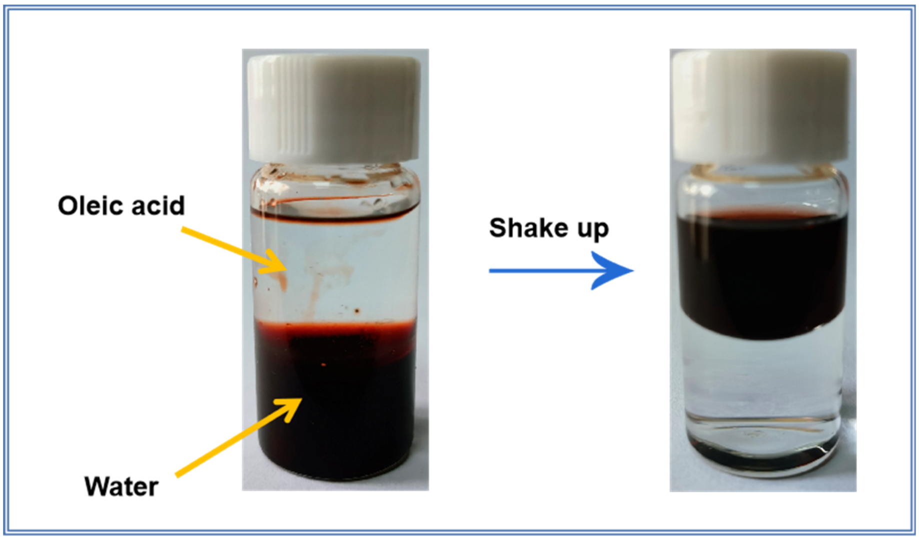

Figure S8. Photographs of **DBPpy** in aqueous media before (left) and after (right) shaking with oleic acid at 37.4℃ for 1h.

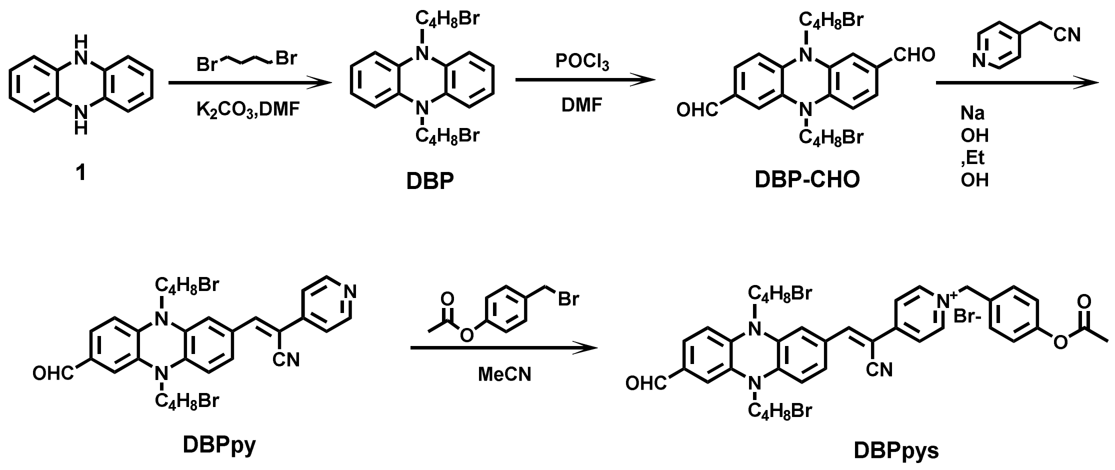

Scheme S1. Synthetic route to the target compound **DBPpys**.

Synthesis of compound DBP.

Compound **1** was prepared through literature reported methods [1-2]. Compound **1** (7.02 g, 36 mmol), K_2CO_3 (7.28 g, 52 mmol) was put into 30 mL DMF, the mixture was heated to 110 °C under argon atmosphere. After stirring for 1 hour, the 1,4-dibromobutane (12 mL, 10 mmol) was added into reaction, then the mixture was stirred for 6 hours. After cooling to room temperature, solvent was removed under vacuum. The crude product was purified by neutral aluminum oxide chromatography, using petroleum ether / dichloromethane (1:1, v/v) as eluent to isolate pure compound **3** (8.2 g, 50.6%) as a green solid. On account of the extreme instability of the product, it was not characterized and quickly put into the next reaction.

Synthesis of DBP-CHO.

Compound **DBP** (5.1g, 9.1 mmol) were dissolved in DMF (15 mL), then added phosphorus oxychloride (2 mL, 21.8 mmol) dropwise under an ice bath with stirring. After addition, it was stirred for 10 minutes and heated to reflux at 120 °C for 8 hours. After the reaction was completed, the mixed solution was cooled and poured into ice water, the pH was adjusted to 9-10 using aqueous NaOH solution. The reaction solution changed from dark red to orange-red and solid was precipitated, filtered and the crude product was then purified by silica gel chromatography using petroleum ether /

dichloromethane (1:5, v/v) as an eluent to isolate pure **DBP-CHO** (2.9 g, 63%) as a red solid. ^{1}H NMR (400 MHz, DMSO-d_6) δ 9.59 (s, 2H), 7.22 (dd, *J* = 8.3, 1.6 Hz, 2H), 6.74 (d, *J* = 1.7 Hz, 2H), 6.55 (d, J = 8.2 Hz, 2H), 3.74 (t, *J* = 6.5 Hz, 4H), 3.53 (t, *J* = 8.0 Hz, 4H), 1.89 (p, *J* = 6.8 Hz, 4H), 1.66 (p, *J* = 7.6, 7.1 Hz, 4H). ^{13}C NMR (101 MHz, Chloroform-d) δ 189.73, 130.18, 110.07, 108.37, 44.25, 30.20, 29.71, 29.47. HRMS (ESI-MS, m/z): Calcd for $[M+H]^+$, 507.0283; Found, 507.0285.

Synthesis of DBPpy.

DBP-CHO (300 mg, 0.56 mmol) and NaOH (24 mg, 0.6 mmol) were added to ethanol and this mixture was stirred and heated at 70 °C for 30 minutes under argon atmosphere. Subsequently, 4-pyridylacetonitrile (66 mg, 0.56 mmol) was added dropwise to the mixture. Then the reaction was quenched with water when its color became deep red. Finally, the product (201 mg, 54.9 %) was purified by silica gel chromatography using dichloromethane / ethyl acetate (5:1, v/v) as an eluent. ^{1}H NMR (400 MHz, DMSO-d_6) δ 9.60 (s, 1H), 8.64 (s, 2H), 8.00 (s, 1H), 7.65 (s, 2H), 7.24 (q, *J* = 7.1, 5.6 Hz, 3H), 6.75 (s, 1H), 6.58 (d, *J* = 8.3 Hz, 2H), 3.74 (q, *J* = 7.1 Hz, 4H), 3.53 (d, *J* = 8.2 Hz, 4H), 1.91 (m, 4H), 1.77-1.63 (m, 4H). ^{13}C NMR (101 MHz, Methylene Chloride-d_2) δ 189.90, 150.79, 144.12, 142.80, 142.64, 140.40, 135.48, 135.30, 130.86, 130.11, 129.91, 126.57, 119.75, 110.75, 110.52, 109.54, 108.67, 103.04, 46.32, 44.92, 29.87, 22.11. HRMS (ESI-MS, m/z): Calcd for $[M+H]^+$, 607.0708; Found, 607.0710.

Synthesis of DBPpys.

DBPpy (103 mg, 0.17 mmol) and 4-bromomethylphenyl acetate (46 mg 0.2 mmol) were added to acetonitrile and this mixture was stirred and heated at 80 °C for 12 hours under argon atmosphere. After removal of the solvent under vacuum, the crude product was purified by silica gel chromatography (dichloromethane / ethanol, 10/1, v/v) gave **DBPpys** (112 mg, 79 %) as a dark green solid. ^{1}H NMR (400 MHz, DMSO-d_6) δ 9.63 (s, 1H), 9.09 (d, *J* = 7.0 Hz, 2H), 8.34 (s, 1H), 8.27 (d, *J* = 6.9 Hz, 2H), 7.61 – 7.54 (m, 2H), 7.36 (dd, *J* = 8.8, 1.9 Hz, 1H), 7.32 – 7.27 (m, 2H), 7.24 – 7.20 (m, 2H), 6.92 –

6.84 (m, 1H), 6.70 – 6.55 (m, 2H), 5.77 (d, *J* = 4.1 Hz, 2H), 3.63 (dt, *J* = 16.3, 6.5 Hz, 4H), 3.53 (t, *J* = 8.5 Hz, 4H), 2.27 (s, 3H), 1.92 (m, 4H), 1.69 (m, 4H). ^{13}C NMR (101 MHz, DMSO-d_6) δ 190.55, 169.60, 151.54, 151.27, 144.62, 142.50, 141.68, 135.15, 134.48, 132.54, 130.71, 130.48, 130.14, 126.14, 123.12, 122.93, 122.65, 120.74, 118.05, 116.16, 111.61, 97.62, 93.47, 61.55, 45.41, 31.61, 30.28, 29.43, 21.30. HRMS (ESI-MS, m/z): Calcd for $[M+H]^+$, 755.1227; Found, 755.1229.

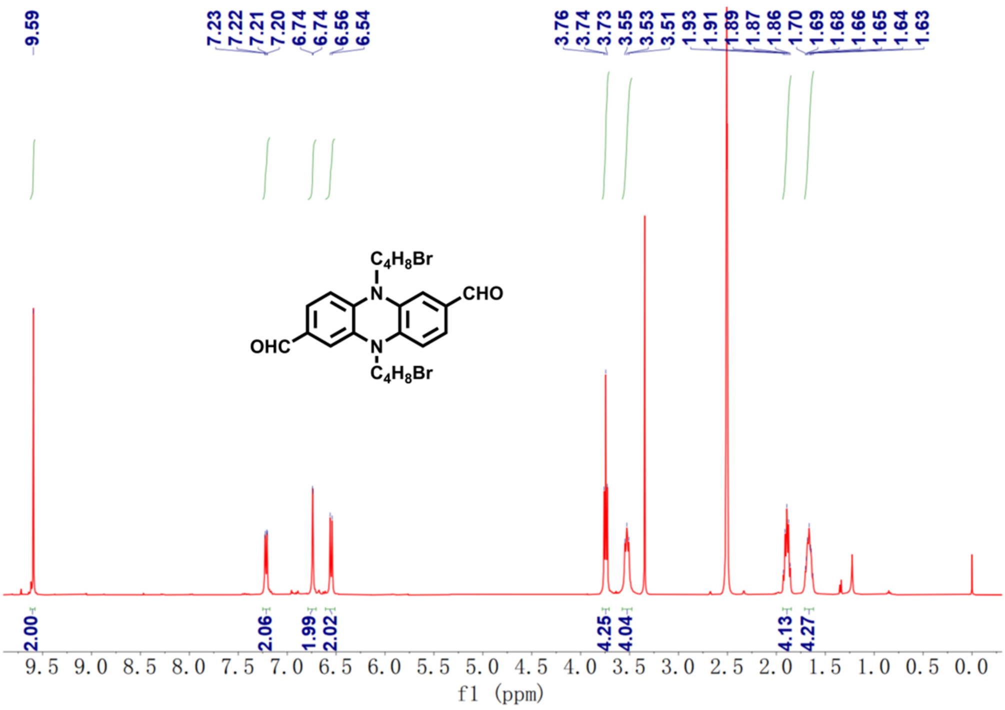

Figure S9. ^{1}H NMR of **DBP-CHO**.

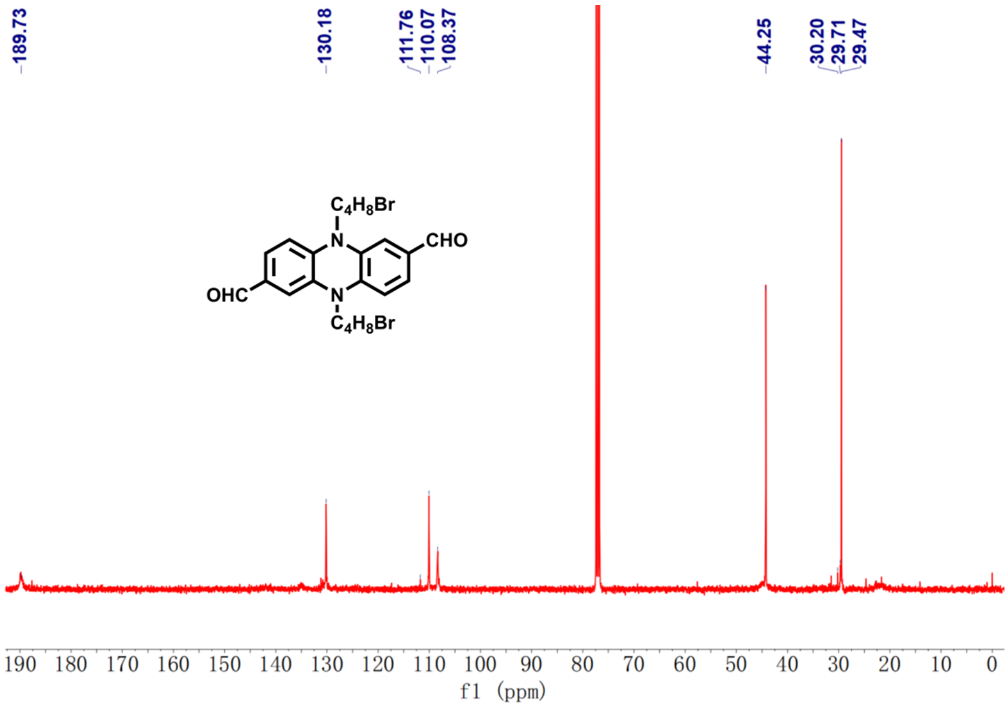

Figure S10. ^{13}C NMR of **DBP-CHO**.

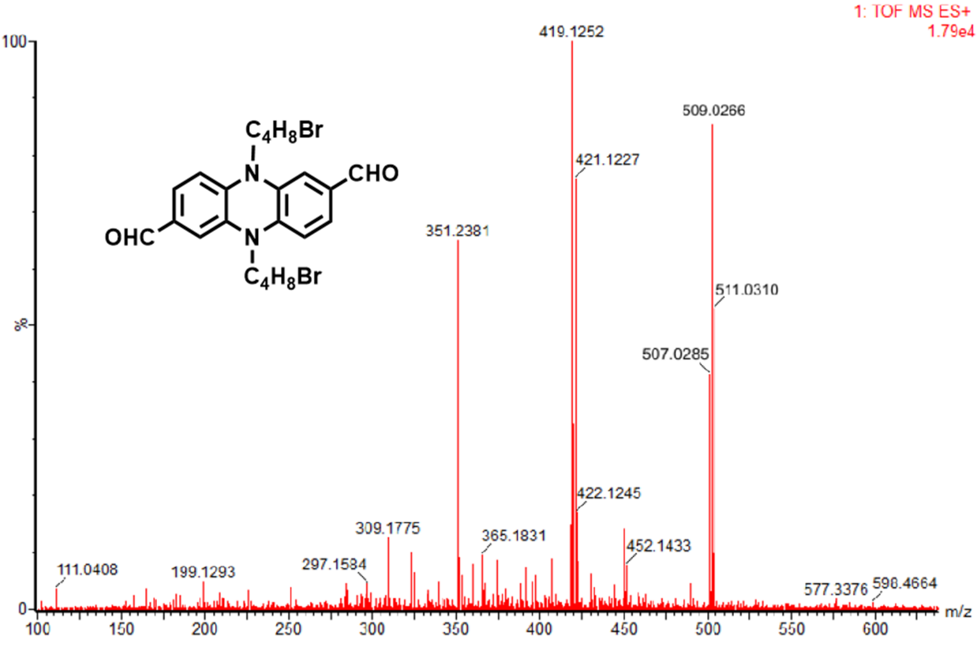

Figure S11. HRMS of **DBP-CHO**.

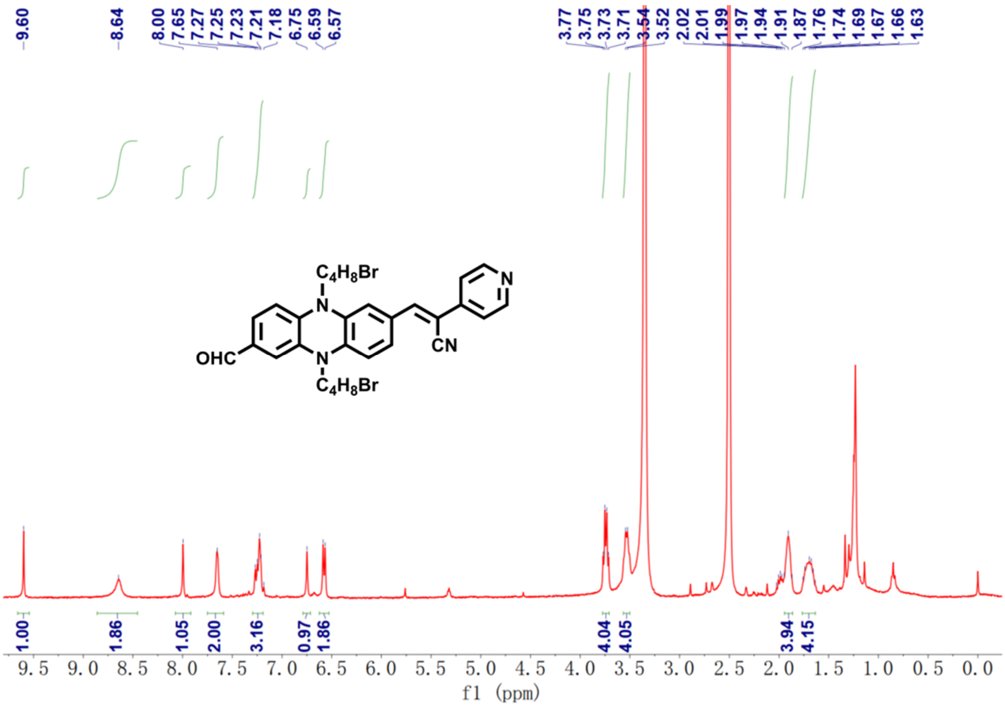

Figure S12. ^{1}H NMR of **DBPpy**.

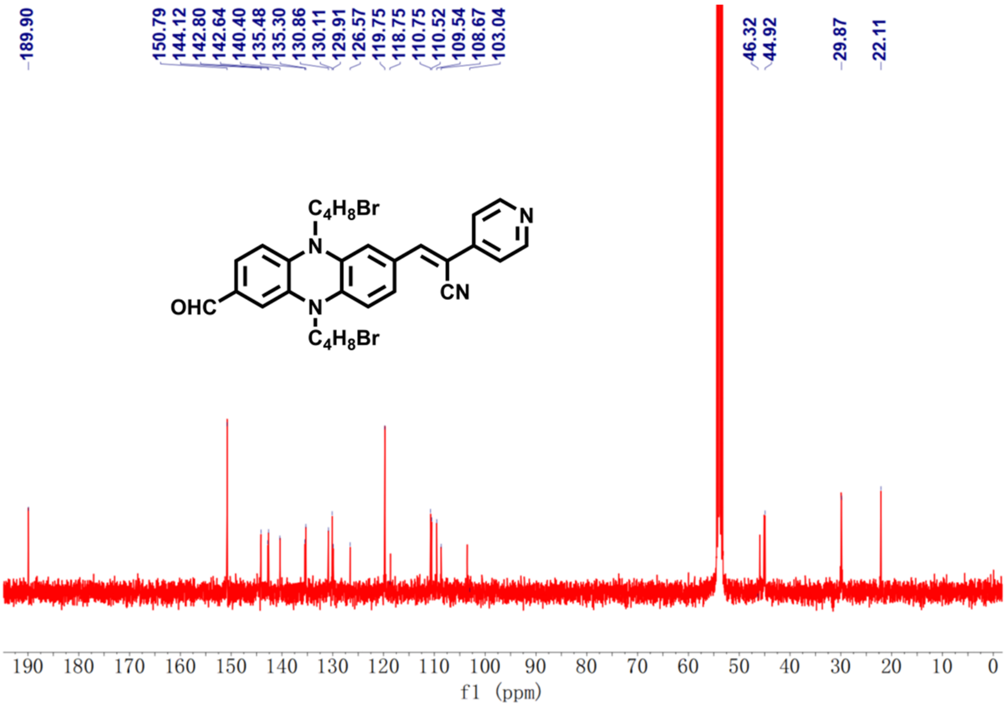

Figure S13. ^{13}C NMR of **DBPpy**.

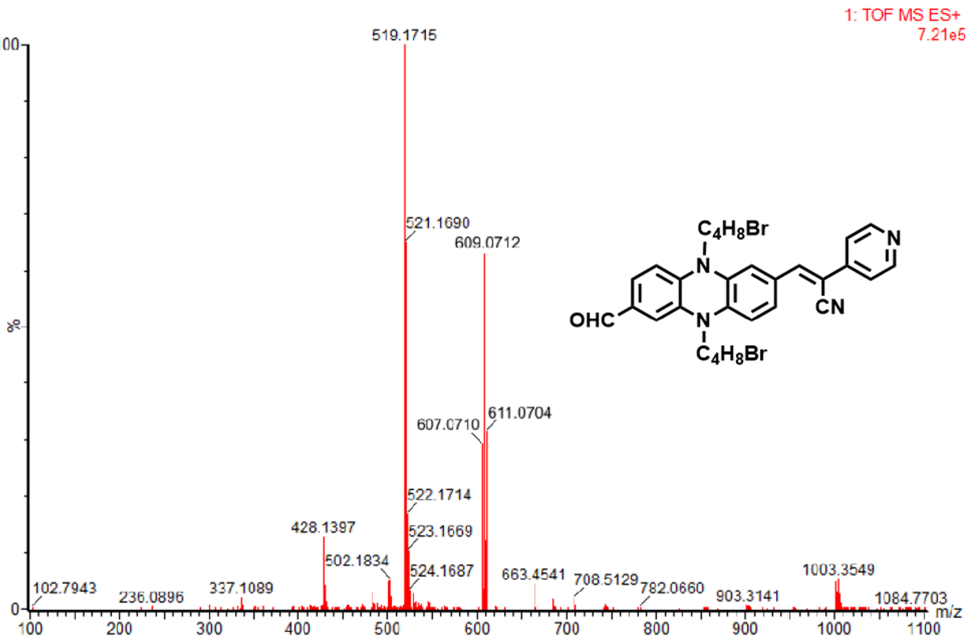

Figure S14. HRMS of **DBPpy**.

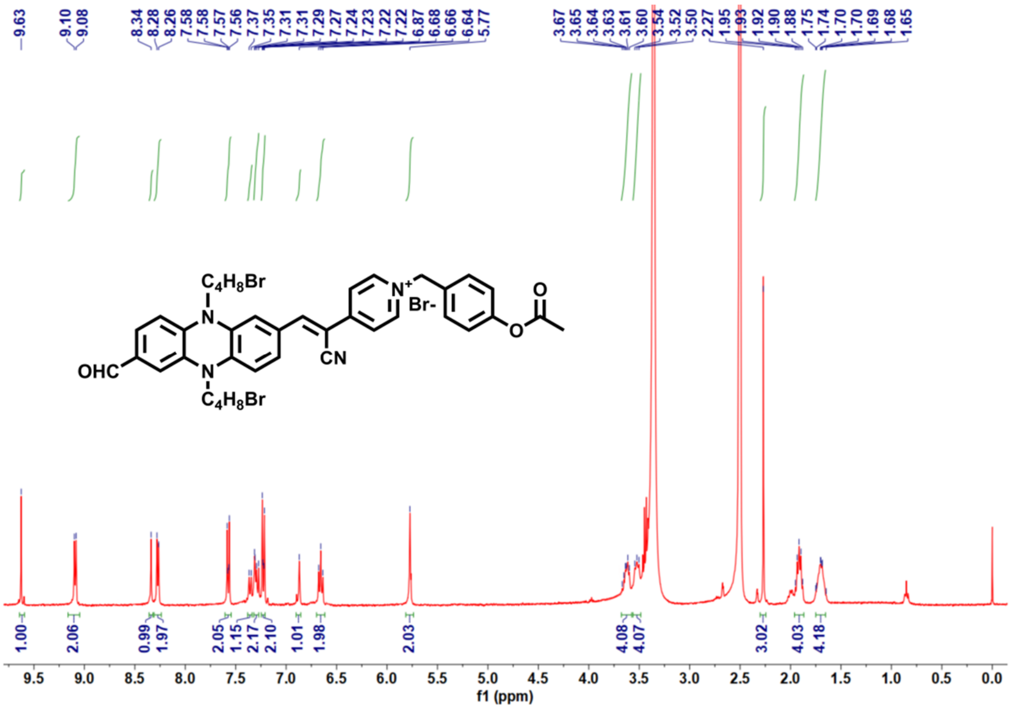

Figure S15. ^{1}H NMR of **DBPpys**.

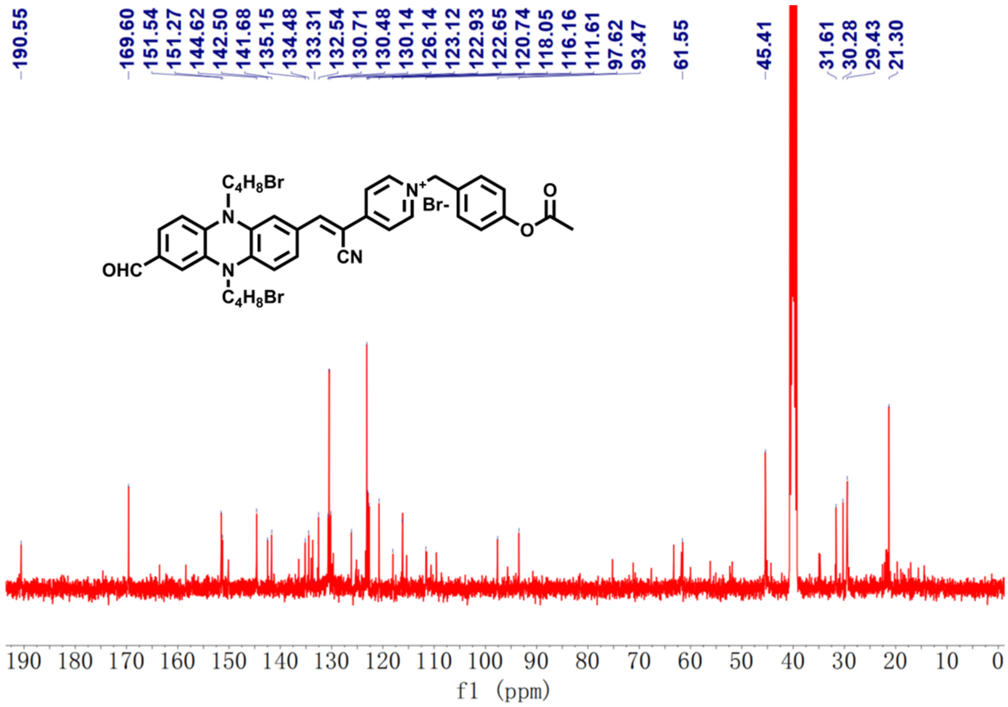

Figure S16. ^{13}C NMR of **DBPpys**.

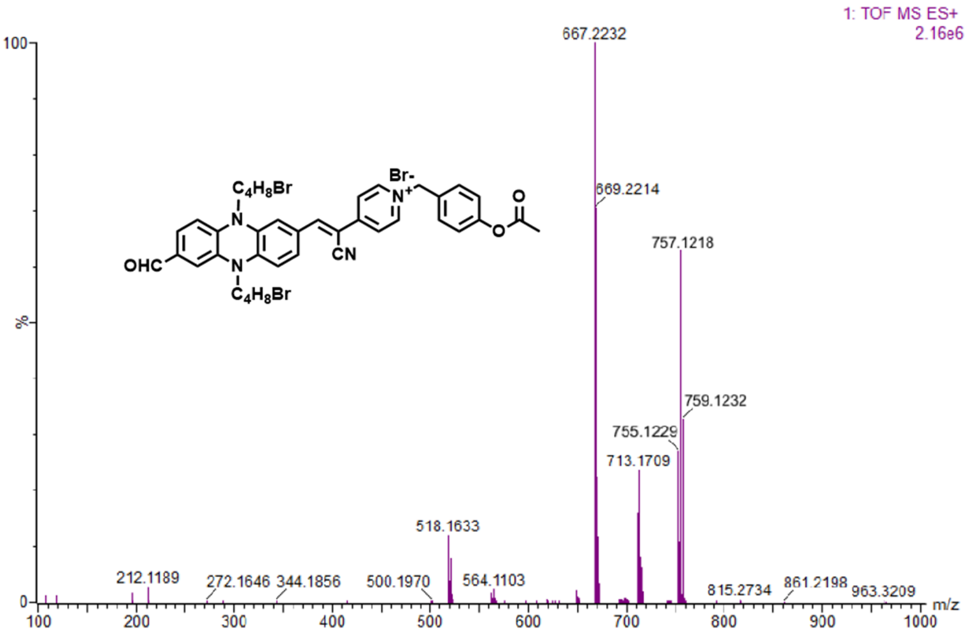

Figure S17. HRMS of **DBPpys**.

References

[1] Yang, L.; Li, X.; Yang, J.; Qu, Y.; Hua, J. Colorimetric and ratiometric near-infrared fluorescent cyanide chemodosimeter based on phenazine derivatives. *ACS Appl. Mater. Inter.* **2013,** *5*, 1317–1326.

[2] Yan, Y.; Liu, L.; Li, C.; Yang, Z.; Yi, T.; Hua, J. A NIR fluorescent probe based on phenazine with a large Stokes shift for the detection and imaging of endogenous H_2O_2 in RAW 264.7 cells. *Analyst* **2020,** *145*, 4196–4203.
